# TMPRSS3 Gene Variants With Implications for Auditory Treatment and Counseling

**DOI:** 10.3389/fgene.2021.780874

**Published:** 2021-11-19

**Authors:** In Seok Moon, Andrew R. Grant, Varun Sagi, Heidi L. Rehm, Konstantina M. Stankovic

**Affiliations:** ^1^ Department of Otolaryngology—Head and Neck Surgery, Massachusetts Eye and Ear and Harvard Medical School, Boston, MA, United States; ^2^ Department of Otorhinolaryngology, Yonsei University College of Medicine, Seoul, Korea; ^3^ Program in Medical and Population Genetics, Broad Institute of MIT and Harvard, Cambridge, MA, United States; ^4^ New York Medical College, Valhalla, NY, United States; ^5^ Department of Otolaryngology—Head and Neck Surgery, Stanford University School of Medicine, Stanford, CA, United States; ^6^ University of Minnesota Medical School, Minneapolis, MN, United States; ^7^ Center for Genomic Medicine and Departments of Pathology and Medicine, Massachusetts General Hospital and Harvard Medical School, Boston, MA, United States

**Keywords:** TMPRSS3, cochlear implantation, sensorineural hearing loss, genetic counseling, hereditary hearing loss

## Abstract

**Objective:** To identify and report novel variants in the *TMPRSS3* gene and their clinical manifestations related to hearing loss as well as intervention outcomes. This information will be helpful for genetic counseling and treatment planning for these patients.

**Methods:** Literature review of previously reported *TMPRSS3* variants was conducted. Reported variants and associated clinical information was compiled. Additionally, cohort data from 18 patients, and their families, with a positive result for *TMPRSS3*-associated hearing loss were analyzed. Genetic testing included sequencing and copy number variation (CNV) analysis of *TMPRSS3* and the Laboratory for Molecular Medicine’s OtoGenome-v1, -v2, or -v3 panels. Clinical data regarding patient hearing rehabilitation was interpreted along with their genetic testing results and in the context of previously reported cochlear implant outcomes in individuals with *TMPRSS3* variants.

**Results:** There have been 87 previously reported *TMPRSS3* variants associated with non-syndromic hearing loss in more than 20 ancestral groups worldwide. Here we report occurrences of known variants as well as one novel variant: deletion of Exons 1–5 and 13 identified from our cohort of 18 patients. The hearing impairment in many of these families was consistent with that of previously reported patients with *TMPRSS3* variants (i.e., typical down-sloping audiogram). Four patients from our cohort underwent cochlear implantation.

**Conclusion:** Bi-allelic variants of *TMPRSS3* are associated with down-sloping hearing loss regardless of ancestry. The outcome following cochlear implantation in patients with variants of *TMPRSS3* is excellent. Therefore, cochlear implantation is strongly recommended for hearing rehabilitation in these patients.

## 1 Introduction

Autosomal recessive non-syndromic hearing loss (ARNSHL) is the most common form of hereditary hearing loss. It accounts for about 70–80% of congenital hereditary hearing loss. ARNSHL is an extremely heterogenous condition as more than 98 loci have been mapped and 77 causative genes have been identified to date (http://hereditaryhearingloss.org/).

The *TMPRSS3* gene encodes a type III transmembrane serine protease that is structurally defined by four functional domains: a transmembrane domain, low density lipoprotein receptor A domain, scavenger receptor cysteine rich domain, and a carboxyl terminal serine protease domain (Südhof et al., 1985; van Driel et al., 1987; Sarrias et al., 2004; Rawlings et al., 2010). The *TMPRSS3* gene is expressed in inner hair cells, spiral ganglion neurons (SGNs), the stria vascularis, and cochlear aqueducts of fetal cochlea (Guipponi et al., 2002). Four alternatively spliced transcripts have been described (DiStefano et al., 2018). The transmembrane serine protease 3 protein is thought to be involved in the development and maintenance of the inner ear, perilymph, endolymph and SGNs (Guipponi et al., 2002). While the function of the *TMPRSS3* gene in the auditory system is not fully understood, its alteration has been linked with non-syndromic genetic hearing loss (DiStefano et al., 2018).

The incidence of *TMPRSS3*-associated ARNSHL is variable amongst different ancestral backgrounds but *TMPRSS3* is a significant contributor in some populations. Pathogenic *TMPRSS3* variants account for 0.7% of Japanese (Miyagawa et al., 2015), 3% of Pakistani (Ben-Yosef et al., 2001), 4.6% of Chinese (Gao et al., 2017), 5–6% of Tunisian (Masmoudi et al., 2001), 5.9% of Korean (Chung et al., 2014), and 11% of Turkish (Wattenhofer et al., 2005) ARNSHL cases. However, this gene has been reported in less than 1% of non-syndromic genetic deafness in White individuals (Wattenhofer et al., 2002). In contrast, pathogenic variants in the *GJB2* gene are found in up to 50% of patients with ARNSHL. Despite the relatively low proportion of ARNSHL cases attributed to *TMPRSS3*, the gene remains a prime candidate for post lingual progressive ARNHSL in North European populations once *GJB2* variants are ruled out (Seligman et al., 2021).

Patients with pathogenic variants in the *TMPRSS3* gene have been described as having one of two discrete hearing phenotypes: severe, prelingual or progressive, post-lingual hearing loss. Weegerink et al. (2011) proposed that the phenotypic outcome of hearing loss is dependent on the combination and severity of *TMPRSS3* variants (i.e., mild or severe). They assert that having two “severe” pathogenic variants leads to profound deafness with prelingual onset (DFNB10), whereas a single ‘severe’ pathogenic variant *in trans* with a milder *TMPRSS3* pathogenic variant yields an initially less severe, but progressive and post-lingual onset hearing loss (DFNB8) (Weegerink et al., 2011). The *TMPRSS3* gene encodes for a transmembrane serine protease which is expressed in SGNs (Guipponi et al., 2002). Therefore, the differential hearing phenotype may reflect the extent of loss of protease activity from a given variant.

In this study, we compile previously reported *TMPRSS3* variants and present a novel variant along with their associated hearing phenotypes. We also aggregate reported outcomes and present new findings regarding the therapeutic effects of cochlear implantation (CI) in patients with pathogenic *TMPRSS3* variants. Together, this information may assist with genetic counseling and treatment planning for patients with *TMPRSS3* variants.

## 2 Methods

### 2.1 Review of the Literature

Literature databases were searched using different combinations of keywords such as “transmembrane serine protease 3,” “TMPRSS3,” “ear,” “hearing loss,” “non-syndromic hearing loss,” and “cochlear implantation.” The databases searched were PubMed, Google Scholar, and two selected gene database websites (https://hereditaryhearingloss.org; https://www.ncbi.nlm.nih.gov/clinvar/). The titles and abstracts were screened using following inclusion criteria: 1) written in English, 2) dealing with non-syndromic hearing loss, and 3) reporting human data.

**FIGURE 1:**
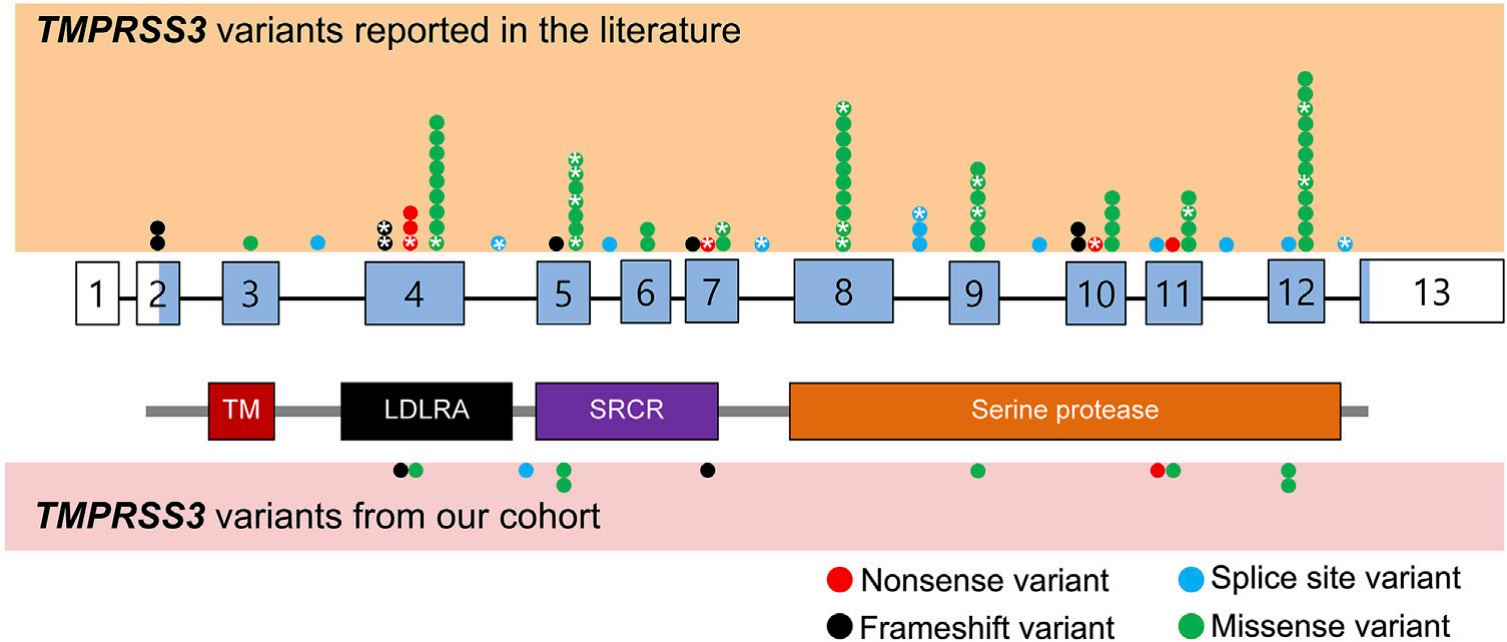
Schematic representation of *TMPRSS3* variants identified from literature review as well as our cohort. Benign variants are not displayed. The exons are numbered with coding sequence shaded blue and untranslated regions unshaded. *: variants with reported cochlear implant outcomes. TM, transmembrane domain; LDLRA, LDL receptor-like domain; SRCR, scavenger receptor cysteine-rich domain.

Based on the search strategy, 39 TMPRSS3-associated papers published from May 2000 to Aug 2021 were reviewed and summarized (Figure 1; Table 1). Among those 39 studies, eleven studies described patients who underwent cochlear implantation (Table 2).

**TABLE 1:** Overview of TMPRSS3 variants resulting in non-syndromic hearing loss, including those identified in the present study.

DNA change	Protein change	Exon	Domain	Variant classification	Origin	Phenotype severity at testing	References
**Deletion of E1-5 and 13**	—	**E1-5 and E13**	—	Pathogenic	**United States**	**Severe**	**This study**
c.36delC	p.Pro12fs	E2			Chinese	Severe	Gao et al. (2017)
c.36dupC	p.Phe13fs	E2			Turkish	—	Diaz-Horta et al. (2012)
c.157G>A	p.Val53Ile	E3	TM		Palestinian	—	Scott et al. (2001)
					Pakistani	—	Ben-Yosef et al. (2001)
					United States		
					Korean	—	Lee et al. (2013)
					Taiwanese	—	Wong et al. (2020)
c.205+38C>T	—	Intron3	—		Taiwanese	—	Wong et al. (2020)
c.207delC	p.Thr70fs	E4			Dutch	—	Ahmed et al. (2004)
					Newfoundlander	—	
					Dutch	—	Weegerink et al. (2011)
c.208delC	p.Thr70fs*19	E4		Pathogenic	Slovenian	Severe	Battelino et al. (2016)
					Polish	—	Lechowicz et al. (2017)
					United States	—	Shearer et al. (2018)
					Slovenian	—	Likar et al. (2018)
					Czech	—	Safka Brozkova et al. (2020)
					**United States**	**Severe**	**This study**
c.212T>C	p.Phe71Ser	E4	LDLRA		Korean	—	Lee et al. (2013)
					Japanese	—	Miyagawa et al. (2015)
c.218G>A	p.Cys73Tyr	E4	LDRLA		Polish	—	Lechowicz et al. (2017)
c.226C>T	p.Gln76X	E4			Japanese	—	Miyagawa et al. (2013)
							Miyagawa et al. (2015)
c.238C>T	p.Arg80Cys	E4	LDRLA	Likely pathogenic	**Europe**	**—**	Capalbo et al., (2019)
					**United States**	**Mild**	**This study**
c.239G>A	p.Arg80His	E4	LDRLA		Taiwanese	—	Wong et al. (2020)
c.268G>A	p.Ala90Thr	E4	LDLRA		UK Caucasian	—	Charif et al. (2012)
					Moroccan		
c.280G>A	p.Gly94Arg	E4	LDLRA		Japanese	—	Miyagawa et al. (2015)
c.296C>A	p.Ser99X	E4			Chinese	Severe	Gu et al. (2015)
c.308A>G	p.Asp103Gly	E4	LDLRA		Greek	—	Wattenhofer et al. (2005)
c.310G>A	p.Glu104Lys	E4	LDLRA		Pakistani	—	Lee et al. (2012)
c.310G>T	p.Glu104X	E4			Pakistani	—	Lee et al. (2012)
c.316C>T	p.Arg106Cys	E4	LDLRA		Japanese	Mild	Miyagawa et al. (2013)
					Chinese		Gao et al. (2017)
c.323-6G>A	—	Intron4	—	Pathogenic	Pakistani	—	Scott et al. (2001)
					Korean	—	Ahmed et al. (2004)
					Dutch	—	Weegerink et al. (2011)
					Chinese	Mild	Gao et al. (2017)
					Pakistani	—	Singh et al. (2020)
					**United States**	**Severe**	**This study**
c.325C>T	p.Arg109Trp	E5	SRCR	Pathogenic	Pakistani	—	Ben-Yosef et al. (2001)
					Pakistani	—	Ahmed et al. (2004)
					Korean	—	Chung et al. (2014)
					Czech	—	Safka Brozkova et al. (2020)
					**United States**	**Mild**	**This study**
c.326G>A	p.Arg109Gln	E5	SRCR		Chinese	—	Gu et al. (2015)
					Polish	Mild	Lechowicz et al. (2017)
c.331G>A	p.Gly111Ser	E5	SRCR		United States	—	Ben-Yosef et al. (2001)
c.346G>A	p.Val116Met	E5	SRCR		Indian	—	Ganapathy et al. (2014)
					Korean	—	Kim et al. (2017)
					Czech	—	Safka Brozkova et al. (2020)
c.371C>T	p.Ser124Leu	E5	SRCR		Polish	—	Lechowicz et al. (2017)
c.390C>G	p.His130Arg	E5	SRCR		Japanese	—	Miyagawa et al. (2015)
c.413C>G	p.Ala138Glu	E5	SRCR	Pathogenic	British	Mild	Weegerink et al. (2011)
					Korean		
					United States	—	Eppsteiner et al. (2012)
					Polish	—	Lechowicz et al. (2017)
					United States	—	Shearer et al. (2018)
					Pakistani	—	Singh et al. (2020)
					**United States**	**Mild**	**This study**
c.432delA	p.Gln144fs	E5			Chinese	—	Sang et al. (2019)
c.447-13A>G	—	Intron 5	—		Pakistani	—	Ben-Yosef et al. (2001)
					United States		
					Taiwanese	—	Wong et al. (2020)
c.453G>A	p.Val151Val	E6	SRCR		Palestinian	—	Scott et al. (2001)
					Pakistani	—	Ben-Yosef et al. (2001)
					United States		
					Korean	—	Lee et al. (2013)
					Taiwanese	—	Wong et al. (2020)
c.551T>C	P.Leu184Ser	E6	SRCR		Chinese	—	Sang et al. (2019)
					Chinese	—	Li et al. (2019)
					Taiwanese	—	Wong et al. (2020)
c.581G>T	p.Cys194Phe	E7	SRCR		Pakistani	—	Ben-Yosef et al. (2001)
						Severe	Ahmed et al. (2004)
c.579dupA	p.Cys194Mfs*17	E7		Pathogenic	Polish	—	Lechowicz et al. (2017)
					**United States**	**Severe**	**This study**
c.595G>A	p.Val199Met	E7	SRCR		Dutch	Severe	Weegerink et al. (2011)
					Korean		
c.607C>T	p.Gln203X	E7			Japanese	Severe	Miyagawa et al. (2013)
c.617-4_-3dupAT	—	Intron7	—		Japanese	—	Miyagawa et al. (2015)
					Taiwanese	—	Wong et al. (2020)
c.621T>C	p.Cys207Cys	E8	Serine protease		Taiwanese	—	Wong et al. (2020)
c.636C>T	p.Gly212Gly	E8	Serine protease		Korean	—	Lee et al. (2013)
c.646C>T	p.Arg216Cys	E8	Serine protease		German	Mild	Elbracht et al. (2007)
					United States (Caucasian)	—	Eppsteiner et al. (2012)
c.647G>T	p.Arg216Leu	E8	Serine protease		Turkish	Severe	Wattenhofer et al. (2005)
					Japanese	—	Miyagawa et al. (2015)
c.726C>G	p.Cys242Trp	E8	Serine protease		Pakistani	Severe	Shafique et al. (2014)
					Czech	—	Safka Brozkova et al. (2020)
c.727G>A	p.Gly243Arg	E8	Serine protease		Indian	—	Ganapathy et al. (2014)
					Pakistani	—	Khan et al. (2019)
c.734C>T	p.Ser245Phe	E8	Serine protease		Czech	—	Safka Brozkova et al. (2020)
c.743C>T	p.Thr248Met	E8	Serine protease		Korean	Mild	Chung et al. (2014)
c.753G>C	p.Trp251Cys	E8	Serine protease		Tunisian	Severe	Masmoudi et al. (2001)
c.757A>G	p.Ile253Val	E8	Serine protease		Pakistani	—	Ben-Yosef et al. (2001)
					United States		
					Korean	—	Lee et al. (2003)
					Taiwanese	—	Wong et al. (2020)
c.767C>T	p.Arg256Val	E8	Serine protease		Pakistani	—	Lee et al. (2012)
c.778G>A	p.Ala260Thr	E8	Serine protease		Japanese	—	Miyagawa et al. (2015)
c.782+8insT	—	Intron8	—		Pakistani	Severe	Ahmed et al. (2004)
c.782+2T>A	—	Intron8	—		Polish	—	Lechowicz et al. (2017)
c.783-1G>A	—	Intron8	—		Korean	—	Kim et al. (2017)
c.809T>A	p.Ile270Asn	E9	Serine protease		Chinese	Severe	Gao et al. (2017)
c.830C>T	p.Pro277Leu	E9	Serine protease		Turkish	—	Masmoudi et al. (2001)
c.871G>C	p.Val291Leu	E9	Serine protease		Korean	—	Lee et al. (2013)
							Kim et al. (2017)
c.916G>A	p.Ala306Thr	E9	Serine protease	Likely pathogenic	German	Severe	Elbracht et al. (2007)
					Dutch	—	Weegerink et al. (2011)
					United States (Caucasian)	—	Eppsteiner et al. (2012)
					Korean	—	Lee et al. (2013)
						—	Chung et al. (2014)
					Tibetan	—	Fan et al. (2014)
					Chinese	—	Gao et al. (2017)
					Korean	—	Song et al. (2020)
					**United States**	**Mild**	**This study**
c.933C>T	p.Ala311Ala	E9	Serine protease		Taiwanese	—	Wong et al. (2020)
c.941T>C	p.Leu314Pro	E9	Serine protease		Pakistani	—	Zhou et al. (2020)
c.953-5A>G	—	Intron 9	—		Polish	—	Lechowicz et al. (2017)
c.974T>A	p.Leu325Gln	E10	Serine protease		Polish	—	Lechowicz et al. (2017)
c.988delA	p.Glu330fs	E10			Pakistani	Severe	Walsh et al. (2006)
c.999delC	p.Asp334Mfs*24	E10			Polish	—	Lechowicz et al. (2017)
c.1019C>G	p.Thr340Arg	E10	Serine protease		Italian	Severe	Vozzi et al. (2014)
c.1025G>A	p.Gly342Glu	E10	Serine protease		Turkish	—	Duman et al. (2011)
c.1028G>C	p.Trp343Ser	E10	Serine protease		Czech	—	Safka Brozkova et al. (2020)
c.1039G>T	p.Glu347X	E10			Korean	—	Song et al. (2020)
c.1128C>T	p.Tyr376Tyr	E11	Serine protease		United States	—	Ben-Yosef et al. (2001)
c.1151T>G	p.Met384Arg	E11	Serine protease		Chinese	Severe	Gao et al. (2017)
c.1156T>C	p.Cys386Arg	E11	Serine protease		Indian	—	Ganapathy et al. (2014)
c.1159G>A	p.Ala387Thr	E11	Serine protease		Japanese	Mild	Miyagawa et al. (2013)
c.1180_1187del8ins68	—	E11	Serine protease		Palestinian	Severe	Scott et al. (2001)
c.1183G>C	p.Asp395His	E11	Serine protease	Unknown	**United States**	**Severe**	**This study**
c.1192C>T	p.Gln398X	E11		Pathogenic	Turkish	Severe	Wattenhofer et al. (2005)
					**United States**	**Severe**	**This study**
c.1194+15C>A	—	Intron 11	—		Taiwanese	—	Wong et al. (2020)
c.1204G>A	p.Gly402Arg	E12	Serine protease		Chinese	Severe	Gao et al. (2017)
					Pakistani	—	Noman et al. (2019)
					United States	—	Bowles et al. (2021)
c.1211C>T	p.Pro404Leu	E12	Serine protease		Tunisian	Severe	Masmoudi et al. (2001)
							Wattenhofer et al. (2005)
					United States	—	Bowles et al. (2021)
c.1219T>C	p.Cys407Arg	E12	Serine protease		Pakistani	Severe	Ben-Yosef et al. (2001)
							Ahmed et al. (2004)
							Lee et al. (2012)
						—	Khan et al. (2019)
						—	Zafar et al. (2020)
c.1244T>C	p.Leu415Ser	E12	Serine protease		Chinese	Severe	Gao et al. (2017)
c.1250G>A	p.Gly417Glu	E12	Serine protease		Chinese	Severe	Gao et al. (2017)
c.1253C>T	p.Ala418Val	E12	Serine protease		Taiwanese	—	Wong et al. (2020)
c.1269C>T	p.Ile423Ile	E12	Serine protease		Taiwanese	—	Wong et al. (2020)
c.1273T>C	p.Cys425Arg	E12	Serine protease		Pakistani	—	Lee et al. (2012)
c.1276G>A	p.Ala426Thr	E12	Serine protease	Likely pathogenic	Dutch	Mild	Weegerink et al. (2011)
					Italian		Leone et al. (2017)
					Polish	—	Lechowicz et al. (2017)
					United States	—	Shearer et al. (2018)
						**Mild**	**This study**
c.1291C>T	p.Pro431Ser	E12	Serine protease		Italian	Severe	Vozzi et al. (2014)
c.1306C>G	p.Arg436Gly	E12	Serine protease	Likely pathogenic	Polish	—	Lechowicz et al. (2017)
					Czech	—	Safka Brozkova et al. (2020)
					**United States**	**Severe**	**This study**
c.1343T>C	p.Met448Thr	E12	Serine protease	Likely pathogenic	Polish	—	Lechowicz et al. (2017)
					Czech	—	Safka Brozkova et al. (2020)
					**United States**	**Mild**	**This study**
c.1345-2A>G	—	E12			United States	—	Shearer et al. (2018)

TM, transmembrane domain; LDLRA, LDL receptor-like domain; SRCR, scavenger receptor cysteine-rich domain; serine protease, trypsin-like serine protease domain. Naming of variants and labeling of domains and exons are based on the NM_001256317.3 transcript. Variant classification based on LMM variant classification. Only predicted loss-of-function and coding variants were included in the table. Bolded text refers to variants identified in this study. Of note, the phenotype severity is provided at the time of testing. While some patients may initially have milder phenotypes, the hearing loss can progress and become more severe.

**TABLE 2:** Overview of clinical characteristics and genotypes of patients with *TMPRSS3* variants who have received cochlear implantation.

Study (country)	DNA change	Protein change	Exon	Domain	Hearing loss severity	Age at CI (gender)	Age at severe-profound HL	Pre-operative hearing	CI type	CI outcomes
Weegerink et al., 2011 (Netherlands)	c.207delC	p.Thr70fs	E4		—	4.5 years	—	Sloping HL	Nucleus Freedom (Cochlear)	91% Phoneme (76% WRS)
	c.916G>A	p.Ala306Thr	E9	Serine protease				40–60–100–110–110 dB (0.25, 0.5, 1, 2, 4 kHz)		
	c.595G>A	p.Val199Met	E7	SRCR	—	6 years	—	Sloping HL	Nucleus Freedom (Cochlear)	80% Phoneme (65% WRS)
	c.916G>A	p.Ala306Thr	E9	Serine protease				40–50–110–110–110 dB (0.25, 0.5, 1, 2, 4 kHz)		
	c.413C>G	p.Ala138Glu	E5	SRCR	—	29 years	—	Decreasing HL	Nucleus CI24M (Cochlear)	—
	c.916G>A	p.Ala306Thr	E9	Serine protease				80–90–100–110–110 dB (0.25, 0.5, 1, 2, 4 kHz); 5% Phoneme		
	c.207delC	p.Thr70fs	E4		—	49 years	—	Decreasing HL	Nucleus Contour CI24R (Cochlear)	89% Phoneme (75% WRS)
	c.1276G>A	p.Ala426Thr	E12	Serine protease				70–95–110–110–110 dB (0.25, 0.5, 1, 2, 4 kHz); 20% Phoneme		
	c.207delC	p.Thr70fs	E4		—	45 years	—	Decreasing HL	Clarion AB-5100H (Advanced Bionics)	76% Phoneme (60% WRS)
	c.1276G>A	p.Ala426Thr	E12	Serine protease				80–90–100–110–120 dB (0.25, 0.5, 1, 2, 4 kHz); 5% Phoneme		
	c.207delC	p.Thr70fs	E4		—	46 years	—	Flat	Clarion AB-5100H (Advanced Bionics)	82% Phoneme (58% WRS)
	c.1276G>A	p.Ala426Thr	E12	Serine protease				100–100–110–120–120 dB (0.25, 0.5, 1, 2, 4 kHz); 0% Phoneme		
	c.207delC	p.Thr70fs	E4		—	43 years	—	Flat	Clarion AB-5100H (Advanced Bionics)	83% Phoneme (62% WRS)
	c.1276G>A	p.Ala426Thr	E12	Serine protease				100–90–110–120–120 dB (0.25, 0.5, 1, 2, 4 kHz); 0% Phoneme		
	c.413C>G	p.Ala138Glu	E5	SRCR	—	51 years	—	Decreasing HL	Nucleus Contour CI24R (Cochlear)	88% Phoneme (68% WRS)
	c.595G>A	p.Val199Met	E7	SRCR				80–90–100–110–120 dB (0.25, 0.5, 1, 2, 4 kHz); 2.5% Phoneme		
	c.413C>G	p.Ala138Glu	E5	SRCR	—	30 years	—	Sloping HL	Nucleus Freedom (Cochlear)	—
	c.323-6G>A	—	In4	SRCR				50–90–110–110–110 dB (0.25, 0.5, 1, 2, 4 kHz); 10% Phoneme		
Eppsteiner et al., 2012 (United States)	c.413C>G	p.Ala138Glu	E5	SRCR	Mild	45 years (male)	45 years	93 dB	Advanced Bionics CII	Poor performance (Combined CNC & HINT Score: 37)
	c.646C>T	p.Arg216Cys	E8	Serine protease	Mild			(PTA at 0.5, 1, 2, and 4 kHz)		
	c.413C>G	p.Ala138Glu	E5	SRCR	Mild	32 years (female)	17 years	98 dB	Advanced Bionics CII	Poor performance (Combined CNC & HINT Score: 23)
	c.916G>A	p.Ala306Thr	E9	Serine protease	Severe			(PTA at 0.5, 1, 2, and 4 kHz)		
Miyagawa et al., 2013 (Japan)	c.607C>T	p.Gln203X	E7		Severe	40 years (female)	—	Sloping HL	MED-EL Pulsar FLEXeas	40–35–30–35–40–40–45 dB (0.125, 0.25, 0.5, 1, 2, 4, 8 kHz)
	c.1159G>A	p.Ala387Thr	E11	Serine protease	Mild			25–30–65–100–110–110–100 dB (0.125, 0.25, 0.5, 1, 2, 4, 8 kHz)		
Chung et al., 2014 (Korea)	c.325C>T	p.Arg109Trp	E5	SRCR	—	12 years (female)	—	Flat (<sloping)	—	Mean open set sentence score at 6 months following CI was 88.5%
	c.916G>A	p.Ala306Thr	E9	Serine protease	—			100–110–110–110–110–110 dB (0.25, 0.5, 1, 2, 4, 8 kHz)		
	c.325C>T	p.Arg109Trp	E5	SRCR	—	6 years (male)	—	Decreasing HL	—	Mean open set sentence score at 6 months following CI was 88.5%
	c.916G>A	p.Ala306Thr	E9	Serine protease	Profound			70–80–90–100–110–100 dB (0.25, 0.5, 1, 2, 4, 8 kHz)		
Miyagawa et al., 2015 (Japan)	c.390C>G	p.His130Arg	E5	SRCR	—	45 years (male)	—	Sloping HL	MED-EL PULSAR FLEX24	90% discrimination score on Japanese monosyllable test at 24 months
	c.647G>T	p.Arg216Leu	E8	Serine protease	—			25–30–65–100–110–110–100 dB (0.125, 0.25, 0.5, 1, 2, 4, 8 kHz); 30% WRS w/HA		
	c.226C>T	p.Gln76X	E4		—	39 years (female)	—	Flat (<-Sloping)	MED-EL PULSAR FLEX24	70% discrimination score on Japanese monosyllable test at 12 months
	c.778G>A	p.Ala260Thr	E8	Serine protease	—			70–90–100–100–110–110–100 dB (0.125, 0.25, 0.5, 1, 2, 4, 8 kHz); 24% WRS w/HA		
	c.212T>C	p.Phe71Ser	E4	LDLRA	—	51 years (female)	—	Sloping HL	MED-EL PULSAR FLEX24	80% discrimination score on Japanese monosyllable test at 12 months
	c.617-4_-3dupAT	p.Thr205fs	In7	—	—			30–40–40–40–100–110–100 dB (0.125, 0.25, 0.5, 1, 2, 4, 8 kHz); 40% WRS w/HA		
Battelino et al., 2016 (Slovenia)	c.208delC^a^	p.Thr70fs^a^19	E4		—	11 months (male)	—	80–110 dB (unclear methodology)	—	25 dB (unclear methodology)
	c.208delC^a^	p.Thr70fs^a^19	E4		—	30 months (male)	—	95–110 dB (unclear methodology)	—	45 dB (unclear methodology)
	c.208delC^a^	p.Thr70fs^a^19	E4		—	13 months (male)	—	80–100 dB (unclear methodology)	—	25 dB (unclear methodology)
	c.208delC^a^	p.Thr70fs^a^19	E4		—	11 months (male)	—	70–85 dB (unclear methodology)	—	25 dB (unclear methodology)
Gao et al., 2017 (China)	c.916G>A	p.Ala306Thr	E9	Serine protease	Severe	3 years (female)	—	Decreasing HL	—	Described as “improved”
	c.1250G>A	p.Gly417Glu	E12	Serine protease	Severe			60–80–80–100–100 dB (0.25, 0.5, 1, 2, 4 kHz)		
	c.916G>A	p.Ala306Thr	E9	Serine protease	Severe	14 years (female)	—	Sloping HL	—	Described as “improved”
	c.323-6G>A	—	In4	—	Severe			20–20–60–100–100–100 dB (0.25, 0.5, 1, 2, 4 kHz)		
Kim et al., 2017 (Korea)	c.346G>A	p.Val116Met	E5	SRCR	—	4 years (female)	—	Decreasing HL	—	Not described, unofficially good
	c.783-1G>A	—	In8	—	Uncertain			90–100–100–1,100–110 dB (0.25, 0.5, 1, 2, 4 kHz)		
	c.346G>A	p.Val116Met	E5	SRCR	Profound	10 years (female)	—	Sloping HL	—	Not described, unofficially good
	c.871G>C	p.Val291Leu	E9	Serine protease	Uncertain			45–90–100–100–110 dB (0.25, 0.5, 1, 2, 4 kHz)		
Shearer et al., 2018 (United States)	c.208delC	p.Thr70fs^a^19	E4		—	64 years	—	—	Nucelus Hybrid CI L24 Array	80–90–110–110–110 dB (0.125, 0.25, 0.5, 1, 2 kHz)
	c.1276G>A	p.Ala426Thr	E12	Serine protease						
	c.413C>G	p.Ala138Glu	E5	SRCR	—	53 years	—	—	Nucleus Hybrid CI S8 Array	50–60–90–110–110 dB (0.125, 0.25, 0.5, 1, 2 kHz)
	c.1276G>A	p.Ala426Thr	E12	Serine protease	—					
	c.1345–2A>G^a^	—	In12	—	—	38 years	—	—	Nucelus Hybrid CI L24 Array	35–30–55–110–110 dB (0.125, 0.25, 0.5, 1, 2 kHz)
Song et al., 2020 (Korea)	c.916G>A	p.Ala306Thr	E9	Serine protease	—	17 years (female)	3–5 years	Sloping HL	—	86% WRS at 12 months following implantation
	c.1039G>T	p.Glu347Ter	E10	Serine protease	—			40–90–100–100–110–110 dB (0.25, 0.5, 1, 2, 4, 8 kHz)		
Holder et al., 2021 (United States)	c.208delC	p.Thr70fs^a^19	E4		—	54 months (female)	—	Sloping HL	Cochlear Nucleus 522/532 (left/right)	CNC 84%; BabyBio Quiet 94%/92% (left/right)
	c.916G>A	p.Ala306Thr	E9	Serine protease				20–25–95–110–100 dB (0.25, 0.5, 1, 2, 4 kHz)		
	c.208delC	p.Thr70fs^a^19	E4		—	47 months (female)	—	Sloping HL	Cochlear Nucleus 522/522 (left/right)	CNC 72%; BabyBio Quiet 55%
	c.916G>A	p.Ala306Thr	E9	Serine protease				20–20–75–115–115 dB (0.25, 0.5, 1, 2, 4 kHz)		
	c.208delC	p.Thr70fs^a^19	E4		—	43 months (female)	—	Sloping HL	Cochlear Nucleus 532/532 (left/right)	LNT 92%/82% (left/right); HINT 62%
	c.916G>A	p.Ala306Thr	E9	Serine protease				20–25–15–95–110 (0.25, 0.5, 1, 2, 4 kHz)		

HL, hearing loss; CI, cochlear implant; LDLRA, LDL receptor-like domain; dB, decibel; WRS, word-recognition score; SRCR, scavenger receptor cysteine-rich domain; serine protease, trypsin-like serine protease domain; PTA, pure tone average; CNC, consonant-nucleus-consonant; HINT, hearing in noise test; HA, hearing aid; LNT, lexical neighborhood test. Naming of variants and labeling of domains and exons are based on the NM_001256317.3 transcript. Of note, the phenotype severity is provided at the time of testing. While some patients may initially have milder phenotypes, the hearing loss can progress and become more severe.

a Patient is homozygous for the specified variant.

Previously reported variants and their associated hearing phenotypes and clinical outcomes following CI, when available, were compiled. Additionally, our own cohort of patients was genetically screened as described below.

### 2.2 Cohort Description

Our study included genetic and phenotypic data from 18 patients and their family members (when available), who were largely White, though Family A was a consanguineous White Egyptian family, Family B was “mixed,” and Families M and I were of Hispanic or Latino ethnicity. Of the patients with characterized hearing loss, the severity ranged from moderate to profound with some individuals experiencing congenital onset and others experiencing a childhood onset or an onset in the second decade of life. Patients were referred to the Laboratory for Molecular Medicine (LMM) at Mass General Brigham Personalized Medicine (Cambridge, MA, United States) from 2009 to 2017. Patients were referred from various clinics and hospitals across the United States. The LMM collected information pertinent to the nature of the hearing loss in the patients (if available) including family history of hearing loss and/or disease, audiological testing, temporal bone CT/MRI results, and CI status. Further information was requested through physicians via the Mass General Brigham Human Research Committee’s IRB protocol for the study of the genetics of hearing loss. Patients were selected based on whether they received a positive result for *TMPRSS3*-associated hearing loss with the intent of follow up of the outcome of CI, if received.

### 2.3 *TMPRSS3* Screening and OtoGenome Next-Generation Sequencing Testing

Patient DNA was extracted from whole blood from patients who were referred to the LMM for hearing-loss genetic testing. Our cohort contains patients from 2009 to 2017. The genetic testing varied for each patient based on the judgment of the ordering physician and the nature of the patient’s hearing loss. Testing was performed by single gene sequencing that included *TMPRSS3*, or LMM’s OtoGenome-v1,-v2, or -v3 panels.

The LMM’s bioinformatics pipeline for targeted next generation sequencing (NGS) panels has been described previously (Pugh et al., 2016). Patients with hearing loss who underwent genetic testing between 2010 and 2014 were tested with the Otogenome-v1 which included the following 71 genes: *ACTG1*, *ATP6V1B1*, *BSND*, *CCDC50*, *CDH23*, *CLDN14*, *CLRN1*, *COCH*, *COL11A2*, *CRYM*, *DFNA5*, *DFNB31*, *DFNB59*, *DIAPH1*, *ESPN*, *ESRRB*, *EYA1*, *EYA4*, *GIPC3*, *GJB2*, *GJB3*, *GJB6*, *GPR98*, *GPSM2*, *GRHL2*, *GRXCR1*, *HGF*, *ILDR1*, *KCNE1*, *KCNQ1*, *KCNQ4*, *LHFPL5*, *LOXHD1*, *LRTOMT*, *MARVELD2*, *MIR183*, *MIR96*, *MSRB3*, *MTRNR1 (12S rRNA)*, *MTTS1 (tRNAser(UCN))*, *MYH14*, *MYH9*, *MYO15A*, *MYO1A*, *MYO3A*, *MYO6*, *MYO7A*, *OTOA*, *OTOF*, *PCDH15*, *PDZD7*, *POU3F4*, *POU4F3*, *PRPS1*, *RDX*, *SERPINB6*, *SLC17A8*, *SLC26A4 (PDS)*, *SLC26A5*, *TECTA*, *TIMM8A*, *TJP2*, *TMC1*, *TMIE*, *TMPRSS3*, *TPRN*, *TRIOBP*, *USH1C*, *USH1G*, *USH2A*, and *WFS1*.

OtoGenome-v2 was used in patients who underwent testing at the LMM from 2014 to 2015. For this iteration, *PDZD7* and *SLC26A5* genes were removed and the *STRC* gene was added. In addition, copy number variant (CNV) detection was added using VisCap as previously described (Pugh et al., 2016; Tayoun et al., 2016).

OtoGenome-v3, used from 2015 to 2017, included 87 genes but did not include the following genes included in v2: *CRYM*, *GJB3*, *MIR182*, *MYO1A*, *SLC17A8*, and *TJP2.* The following 23 genes were added *CACNA1D*, *CATSPER2*, *CEACAM16*, *CIB2*, *CLPP*, *DIABLO*, *EDN3*, *EDNRB*, *HARS2*, *HSD17B4*, *KARS*, *LARS2*, *MITF*, *OTOG*, *OTOGL*, *P2RX2*, *PAX3*, *SIX1*, *SMPX*, *SOX10*, *SYNE4*, *TBC1D24*, and *TSPEAR*. Parents and other unaffected/affected family members, when available, were tested for detected variants. Variants were confirmed *via* Sanger sequencing for single-nucleotide variants (SNVs), or droplet digital PCR for CNVs called by VisCap (Pugh et al., 2016; Tayoun et al., 2016).

### 2.4 LMM Variant Classification

The LMM’s early variant classification methods are as previously described (Duzkale et al., 2013) and were subsequently updated to conform to more recent professional guidelines (Richards et al., 2015). Data used to classify variants included that from population databases (e.g., Exome Aggregation Consortium (ExAC); gnomAD), internal or external disease databases (e.g., ClinVar, LOVD, HGMD), the literature, functional studies, segregation, allelic observations and *in silico* missense and splicing prediction tools. Variants were classified as pathogenic (P), likely pathogenic (LP), of uncertain significance (VUS), likely benign, or benign. The VUS category was further subdivided into VUS-5, -4, and -3 where VUS-5 indicated leaning towards pathogenic, and VUS-3 indicated leaning towards benign. Likely benign and benign variants are not reported in this article but were submitted to ClinVar (www.ncbi.nlm.nih.gov/clinvar/) along with all other variants observed at the LMM.

## 3 Results

We reviewed the type, position, origin, and variant classification of 87 previously reported *TMPRSS3* variants and present one novel variant identified from our cohort (Figure 1; Table 1). Compiled variants are associated with non-syndromic hearing loss in more than 20 ancestral groups worldwide. Fourteen of the identified variants were predicted loss-of-function (pLOF) (frameshift, stop-codon, or splice-site variants) with either prematurely terminated protein products or nonsense-mediated decay of mRNA. Fifty-eight of the identified variants were missense variants. Nearly all variants were predicted to disrupt the proteolytic activity of the protein. Both prelingual and post lingual hearing impairment was reported, with most patients showing a typical ski-slope audiogram configuration. CI outcomes were reported for 32 patients with bi-allelic variants in *TMPRSS3* across 11 different studies (Table 2) (Weegerink et al., 2011; Eppsteiner et al., 2012; Miyagawa et al., 2013; Chung et al., 2014; Miyagawa et al., 2015; Battelino et al., 2016; Gao et al., 2017; Kim et al., 2017; Shearer et al., 2018; Song et al., 2020; Holder et al., 2021). While degree of hearing improvement varied between patients, the majority of those who underwent CI had positive outcomes.

**FIGURE 2:**
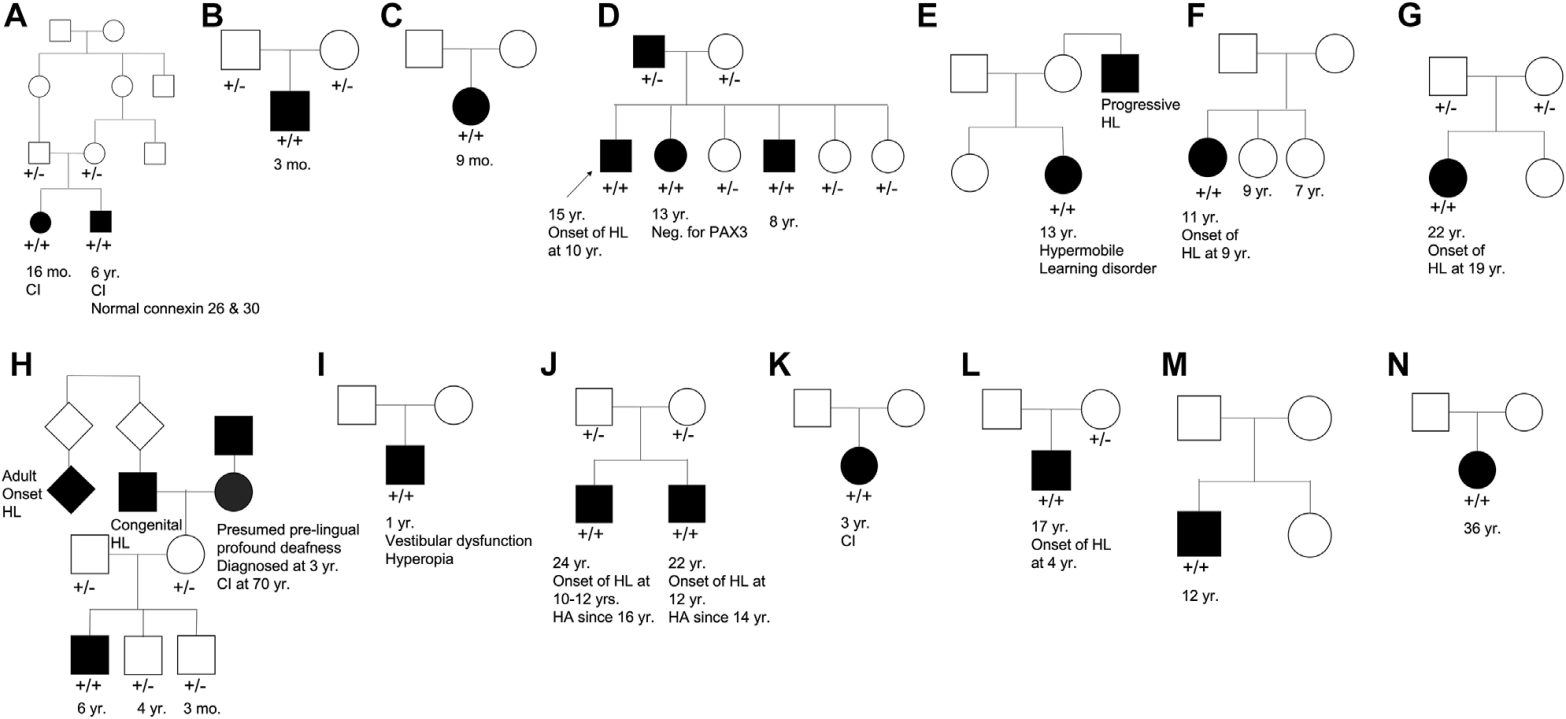
Pedigree chart for enrolled patient families **(A–N)**. Age at genetic testing, age at onset of hearing loss, and other relevant clinical information is provided, when available, for patients and family members. CI, cochlear implant; HL, hearing loss; HA, hearing aid.

Our cohort included 18 patients—7 females and 11 males—with ages ranging from 3 months to 36 years (Figure 2). 15 patients were White with the remaining 3 identifying as Hispanic/Latino or mixed. We identified 12 different *TMPRSS3* variants of which 1 has not been previously reported: deletion of Exons 1–5 and 13 (Table 3). This novel variant was classified as *pathogenic* as it met the criteria outlined by previous professional guidelines (Richards et al., 2015) with specifications provided by ClinGen (https://clinicalgenome.org/working-groups/sequence-variant-interpretation), specifically the combination of PVS1 (predicted loss of function), PM2 (absence in gnomAD), and PM3 (homozygous observation in an individual with phenotype matching the gene). The most commonly identified variants were p.Thr70fs*19 and p.Ala138Glu. Eight patients had congenital hearing loss, four of whom had biallelic pLOF variants.

**TABLE 3:** Genotype and phenotype overview of our patient cohort.

Family	Age	Gender	DNA change	Protein change	Configuration	HL onset	HL severity
A	16 months	F	**Deletion of Exons 1–5 and13** ^a^	—	—	Congenital	—
	6 years	M	**Deletion of Exons 1–5 and13** ^a^	—	—	Congenital	—
B	3 months	M	c.208delC^a^	p.Thr70fs^a^19	—	Congenital	Profound
C	9 months	F	c.208delC; c.1192C>T	p.Thr70fs^a^19; p.Gln398X	—	Congenital	Profound
D	8 years	M	c.208delC; c.1276G>A	p.Thr70fs^a^19; p.Ala426Thr	—	—	Sloping hearing loss
	13 years	F			—	—	Sloping sensorineural hearing loss
	15 years	M			Trans	10 years old	Progressive sloping, moderate left, severe right
E	13 years	F	c.208delC; c.413C>G	p.Thr70fs^a^19; p.Ala138Glu	—	—	Progressive, sloping, severe
F	11 years	F	c.208delC; c.413C>G	p.Thr70fs^a^19; p.Ala138Glu	—	9 years old	Sloping, profound
G	22 years	F	c.208delC; c.413C>G	p.Thr70fs^a^19; p.Ala138Glu	—	19 years old	—
H	6 years	M	c.323-6G>A; c.325C>T	-; p.Arg109Trp	—	Congenital	Moderately severe to profound
I	1 year	M	c.579dupA; c.1183G>C	p.Cys194MetfsX17; p.Asp395His	Trans	Congenital	Severe to profound
J	22 years	M	c.238C>T; c.1343T>C	p.Arg80Cys; p.Met448Thr	—	12 years old	Progressive, moderate-severe left, severe right
	24 years	M	c.238C>T; c.1343T>C	p.Arg80Cys; p.Met448Thr	—	10–12 years old	—
K	3 years	F	c.310G>A; c.916G>A	p.Glu104Lys; p.Ala306Thr	—	—	Moderately severe at low frequencies, profound at high frequencies
L	17 years	M	c.325C>T; c.413C>G	p.Arg109Trp; p.Ala138Glu	—	4 years old	Moderate-severe
M	12 years	M	c.413C>G; c.916G>A	p.Ala138Glu; p.Ala306Thr	—	Congenital	Progressive, high frequency, moderate
N	36 years	M	c.208delC; c.1306C>T	p.Thr70fs^a^19; p.Arg436Gly	—	Congenital	Progressive, profound

HL, hearing loss. Novel variant is bolded. Naming of variants is based on the NM_001256317.3 transcript.

a patient is homozygous for the specified variant.

Four patients in our cohort underwent CI, and outcome information was available for two patients. The first patient, from family B, was found to have congenital profound hearing loss and was homozygous for p.Thr70fs*19*.* It is unclear when the patient underwent CI. However, at a follow up at 4 years of age, the patient had functional speech. Clinical records indicated that the patient had ongoing articulation errors and required speech therapy but was able to maintain adequate hearing. The second patient, from family K, was compound heterozygous for p.Glu104Lys and p.Ala306Thr. Clinical records have suggested positive CI outcome for her moderate-profound hearing loss. The remaining two patients who underwent CI were the siblings from family A who both had profound congenital hearing loss and were homozygous for a deletion of Exons 1–5 and 13. Their current hearing status is unknown.

## 4 Discussion

The genotype-phenotype correlations of *TMPRSS3* variants have not been well characterized. It has been previously shown that the frequency of *TMPRSS3*-induced ARSNHL was low in White individuals (Wattenhofer et al., 2002). However, a recent epidemiological study of patients undergoing CI revealed that 10% (13) of patients with positive genetic testing had *TMPRSS3* gene variants (Seligman et al., 2021). As adoption of genetic testing in clinical practice continues to grow, it is important to be aware of common *TMPRSS3* variants and associated phenotypes to best counsel patients.

In our cohort of 18 patients, 15 of whom were White, the most frequently observed variants were p.Thr70fs*19 and p.Ala138Glu implying that those were either hot spots or founder variants. The combination of the p.Thr70fs*19 frameshift variant with a missense variant appeared to cause sloping hearing loss that varied in severity. Biallelic pLOF variants appeared to cause congenital profound hearing loss. This phenotype information is valuable when trying to understand potential patient prognosis based on genetic testing results.

Previous studies on the role of CI in patients with *TMPRSS3* variants have reported variable results. In one study, poor outcomes following CI in patients with *TMPRSS3* variants were attributed to the expression of the *TMPRSS3* gene in SGNs as opposed to other locations in the cochlea such as the membranous labyrinth (Eppsteiner et al., 2012). These authors also suggested that patients with pathogenic *TMPRSS3* variants may have continued loss of SGNs over time which could contribute to ongoing hearing deterioration even after CI. However, recent studies have shown predominantly positive outcomes following CI in patients with *TMPRSS3* variants (Weegerink et al., 2011; Miyagawa et al., 2013; Chung et al., 2014; Miyagawa et al., 2015; Battelino et al., 2016; Gao et al., 2017; Shearer et al., 2018; Song et al., 2020; Holder et al., 2021). This discrepancy might be related to the large duration of deafness and older age of the two patients in Eppsteiner et al. (2012) and Holder et al. (2021). In addition, a study of CI outcomes in pediatric patients with *TMPRSS3* variants reported positive outcomes with no evidence of SGN degeneration leading to decreased performance over time (Holder et al., 2021). Furthermore, it was suggested that even if SGN degeneration does contribute to a longitudinal decline in performance, early CI may help slow or reverse this process (Holder et al., 2021). Even so, many clinics do not implant patients with precipitously sloping hearing loss as they do not meet *labeled* indications for CI. However, *off-label* implantation has been shown to be beneficial and is being employed much more frequently at major academic medical centers (Carlson et al., 2015; Leigh et al., 2016; Carlson et al., 2018).

Taken together with the positive clinical outcomes following CI in two patients from our cohort, it is evident that CI is a promising treatment strategy for patients with *TMPRSS3* variants. Active intervention with CI is likely to be beneficial, particularly in patients in whom residual hearing is preserved. It is imperative that the benefits of CI are made clear when counseling patients on their potential treatment options.

## Data Availability

The evidence for all variants classified by the authors is included in submissions to ClinVar by the Laboratory for Molecular Medicine (Organization ID: 21766). All other data supporting the conclusions of this article, if not directly included in the paper, will be made available by the authors, without undue reservation.
